# Mechanical Vertebral Body Augmentation Versus Conventional Balloon Kyphoplasty for Osteoporotic Thoracolumbar Compression Fractures: A Systematic Review and Meta-Analysis of Outcomes

**DOI:** 10.1177/21925682241261988

**Published:** 2024-06-18

**Authors:** Matthew Macciacchera, Jake M. McDonnell, Aisyah Amir, Aubrie Sowa, Gráinne Cunniffe, Stacey Darwish, Ciara Murphy, Joseph S. Butler

**Affiliations:** 1 School of Medicine, 655732 Royal College of Surgeons in Ireland, Dublin, Ireland; 2 National Spinal Injuries Unit, 8881 Mater Misericordiae University Hospital, Dublin, Ireland; 3 Trinity Centre for Biomedical Engineering, Trinity Biomedical Sciences Institute, 8809 Trinity College Dublin, The University of Dublin, Dublin, Ireland; 4 School of Medicine, 8797 University of College Dublin, Belfield, Dublin, Ireland; 5 Department of Anatomy and Regenerative Medicine, 655732 Royal College of Surgeons in Ireland, Dublin, Ireland; 6 Advanced Materials and BioEngineering Research= (AMBER) Centre, 8809 Trinity College Dublin, Ireland

**Keywords:** spine surgery, osteoporosis, vertebral body augmentation, comparative, outcomes

## Abstract

**Study Design:**

Systematic review and meta-analysis.

**Objective:**

Surgical management of osteoporotic vertebral compression fractures (OVCFs) has traditionally consisted of vertebroplasty or kyphoplasty procedures. Mechanical percutaneous vertebral body augmentation (MPVA) systems have recently been introduced as alternatives to traditional methods. However, the effectiveness of MPVA systems vs conventional augmentation techniques for OVCFs remains unclear. This serves as the premise for this study.

**Methods:**

A systematic review and meta-analysis was conducted as per the *Preferred Reporting Items for Systematic Reviews and Meta-Analyses (PRISMA)* guidelines. Studies of interest included randomized controlled trials (RCTs) which directly compared patient outcomes following kyphoplasty to patients treated with MPVA systems. Clinical and radiological findings were collated and compared for significance between cohorts.

**Results:**

6 RCTs were identified with 1024 patients total. The mean age of all patients was 73.5 years. 17% of the cohort were male, 83% were female. 515 patients underwent kyphoplasty and 509 underwent mechanical vertebral body augmentation using MPVA systems. MPVAs showed similar efficacy for restoration of vertebral body height (*P* = .18), total complications (*P* = .36), cement extravasation (*P* = .58) and device-related complications (*P* = .06). MPVAs also showed reduced rates of all new fractures (16.4% vs 22.2%; *P* = .17) and adjacent fractures (14.7% vs 18.9%; *P* = .23), with improved visual analogue scale (VAS) scores at 6-month (*P* = .13).

**Conclusion:**

The results of this meta-analysis highlight no significant improvement in clinical or radiological outcomes for MPVA systems when compared to balloon kyphoplasty for vertebral body augmentation. Further research is needed to establish a true benefit over traditional operative methods.

## Introduction

Osteoporosis is an increasingly common skeletal disorder characterised by bone mineral loss and decreased bone strength. The reported prevalence of osteoporosis worldwide is 18.3%,^1^ with a 50% lifetime risk of osteoporotic fracture in women and 25% in men.^2^ The most common form of osteoporotic fractures are vertebral compression fractures (OVCFs). Patients with OVCFs may be successfully treated through conservative management consisting of pain medication, bed rest, bracing and physiotherapy with the main focus of these interventions being spinal support and symptom alleviation. However, it is well described in the literature that conservative therapy does not ultimately correct spinal misalignment. If symptoms persist and fracture healing fails to occur, patients are at risk of kyphotic deformity leading to impaired physical function and reduced quality of life.^3^ The surgical techniques vertebroplasty and kyphoplasty were developed as alternatives to conservative management to reduce patient morbidity. While these procedures often restore function and reduce pain, major disadvantages include incomplete fracture reduction and loss of height restoration through cement leakage.^4^

Mechanical percutaneous vertebral body augmentation (MPVA) systems have recently been introduced as a novel surgical treatment option for spinal surgeons to consider when managing OVCFs. The Kiva® system is a non-biodegradable implant designed to direct the flow of cement, which has been shown to require low intra-operative cement volumes.^4^ The SpineJack® system, another non-biodegradable implant, is used to effectively restore and maintain vertebral height before cement injection.^5^ The KyphX Xpander® is an inflatable bone tamp system which is also commonly used in OVCF treatment.^6,7^

However, while the knowledge of these systems are becoming increasingly well known, the effectiveness of these devices in comparison to traditional OVCF surgical techniques remains unclear. The purpose of this study was to review the available literature which compares patient outcomes following balloon kyphoplasty vs the use of MPVA systems to treat osteoporotic compression fractures. Through collative statistics via meta-analyses, this article provides practicing spine surgeons with the evidence necessary for informed decision making in the management of vertebral compression fractures patients with osteoporosis.

## Methods

### Search Strategy and Study Selection

Two independent reviewers (MM and JMM) performed a literature search following the *Preferred Reporting Items for Systematic Reviews and Meta-Analyses (PRISMA)* guidelines.^8^ Any disagreements regarding study inclusion were resolved by consulting the senior author (JSB). A comprehensive search was performed for eligible articles using Pubmed/Medline, Embase and Cochrane databases to include studies up to and including July 27^th^, 2023. Search items included “Percutaneous vertebral body augmentation” AND (“kyphoplasty”) AND (“outcomes” OR “complications”) OR (“morbidity” OR “mortality” OR “revision” OR “reoperation”) AND (“SpineJack” OR “Osseofix” OR “Vertebral Body Stenting” OR “Kiva” OR “iVAS” OR “StabiliT”). Reference lists of full-text articles were reviewed and screened for further studies meeting the inclusion criteria.

### Eligibility criteria

The inclusion criteria for this study were (i) all years, (ii) comparative studies (MPVAs vs kyphoplasty), (iii) English studies (or direct translation available), (iv) must report on patient outcomes, (v) osteoporotic compression fractures, (vi) thoracolumbar region. The exclusion criteria included (i) non-comparative studies (case reports or reviews), (ii) other fracture patterns other than osteoporotic compression fractures, (iii) conservative management, (iv) pathological fractures (patients with spinal metastases).

### Data Extraction

All relevant information was collected by 2 independent reviewers. The *Methodological Quality of Evidence (MQOE)* was assessed using the Risk Of Bias 2 (RoB-2) tool developed by Cochrane for evaluating bias in randomised studies.^9^

### Outcomes Analysed and Statistics

Outcomes analysed were operative characteristics (time, amount of cement used), radiological outcomes (midline vertebral body height ratio), complications (cement extravasation, device related, new/adjacent fractures, total), functional outcomes (Visual Analogue Scale change from baseline at 6 months and final follow-up, Oswestry Disability Index change from baseline at 6 months and final follow-up). Heterogeneity between studies was quantified using the I^2^ statistic. A random effects model and binary effects model were employed. Results are expressed as mean for continuous outcomes and risk ratio (RR) for dichotomous outcomes, with a 95% confidence interval (CI). Heterogeneity values were interpreted per Cochrane values; (i) 0%-40% = low degree of heterogeneity (ii) 30%-60% = moderate degree of heterogeneity (iii) 50%-90% = substantial degree of heterogeneity (iv) 75%-100% = considerable degree of heterogeneity. A *P*-value of <.05 was considered to be statistically significant.

### Aims and Objectives

**Figure 1.**
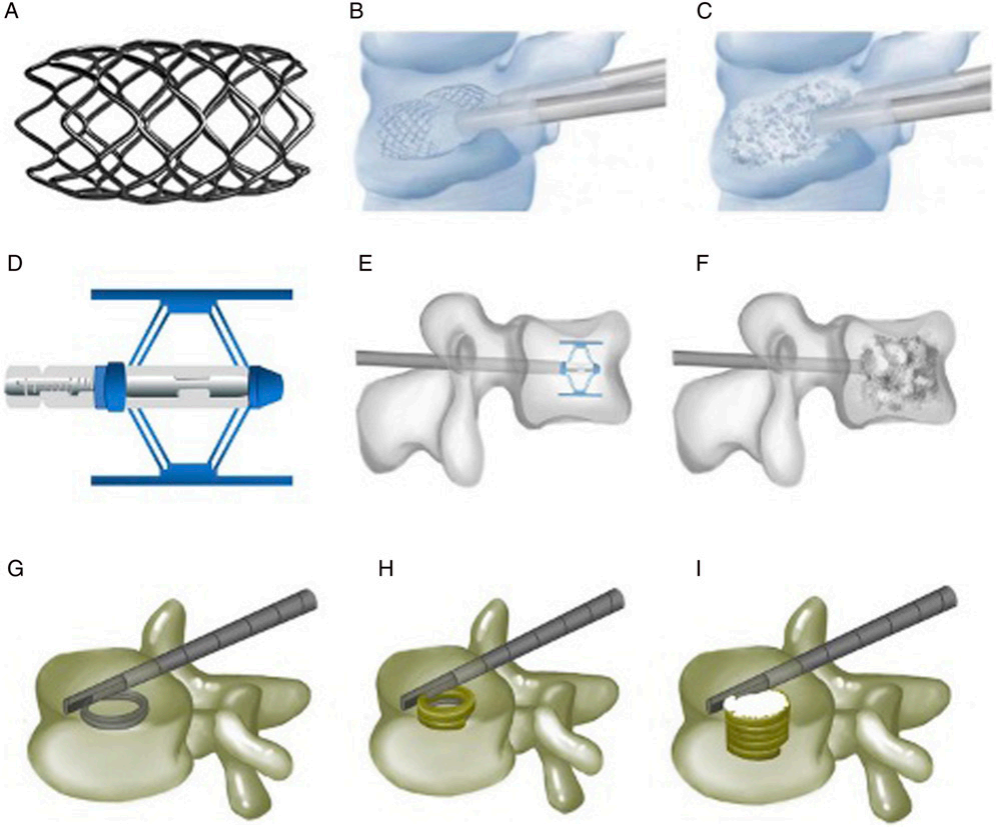
Vertebral body stenting, Spine Jack®, and Kiva system®. (A) Vertebral body stenting appearance. (B) Vertebral restoration using vertebral body stenting. (C) Bone cements injected after vertebral body stenting expansion. (D) Expanded Spine Jack®. (E) Spine Jack® expanded inside the vertebra via a transpedicular approach. (F) Bone cements augmenting the restored vertebra after the Spine Jack® is placed inside the vertebra. (G) Guided coil of the Kiva® system inserted via transpedicular cannula. (H) PEEK implant of the Kiva® system along the guide wire forming a hollow cylinder inside the vertebra. (I) Injected polymethylmethacrylate cement, surrounded by the Kiva® system. Adapted from Luo et al. 2023(7) under the under the Creative Commons Attribution-Non-Commercial License 4.0 (CCBY-NC).

The aim of this study was to compare patient and procedure related outcomes following vertebral body augmentation using MPVA systems or the kyphoplasty techniques. The objective of this study was to determine whether novel MPVA devices improve post-operative functional outcomes and/or reduce post-operative complications, in osteoporotic patients with vertebral compression fractures (Figure 1).

## Results

**Figure 2.**
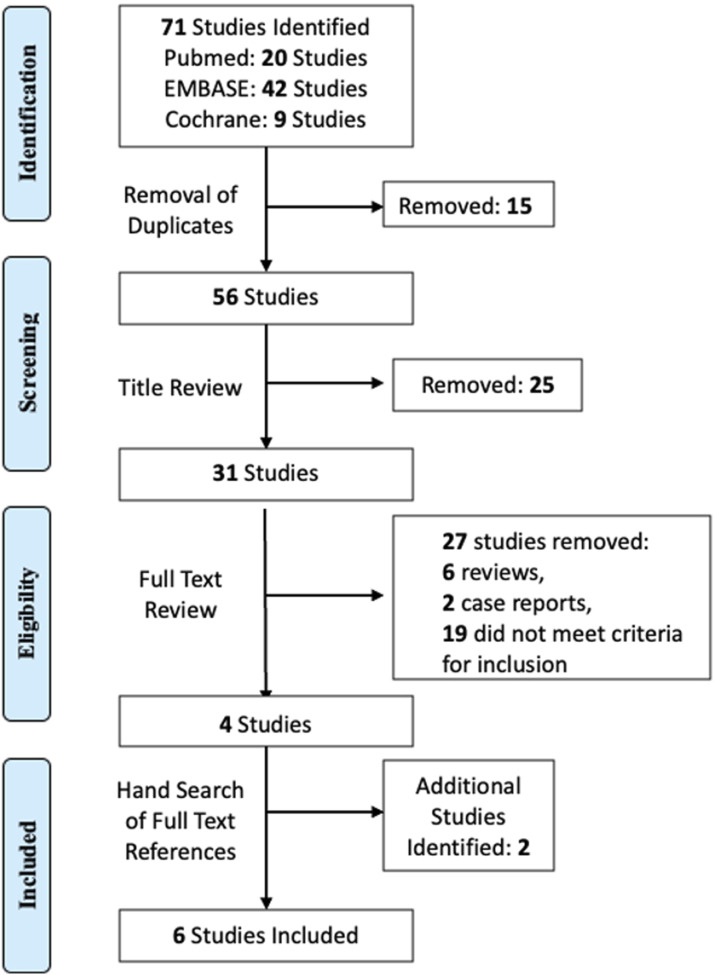
PRISMA flowchart per the *Preferred Reporting Items for Systematic Reviews and Meta-Analyses (PRISMA)* guidelines.

The literature search yielded 71 results (Figure 2). After removal of duplicates, 56 articles remained for screening of title and abstract. 25 articles were subsequently excluded, leaving 31 for full-text review. 4 studies met the predefined inclusion criteria and were considered for full-text review. 2 additional studies were identified using article reference sections. Thus, 6 studies were included for literature review and data synthesis through meta-analysis.

Overall, there were 1024 patients included across the 6 studies. 515 patients underwent kyphoplasty and 509 underwent mechanical vertebral body augmentation using MPVA systems (Kiva®/Spinejack®/Xpander®/BKP + Spinejack®). The mean age of all patients was 73.5 years. Sex was defined in 5 studies. Of those, 17% of the cohort were male, 83% were female. All studies included patient populations with osteoporosis. Study characteristics are outlined in Table 1.

**Table 1.** Characteristics of studies. (BKP) = Balloon Kyphoplasty.

Author	Year	Sample Size (BKP)	Sample Size (MPVA)	Mean Age	Male:Female	Final Follow-up
Korovessis et al^4^	2013	86 (122 vertebrae)	82 (133 vertebrae)	70.5	49:119	14 months
Noriega et al^2^	2016	15	15	68	6:24	12 months
Noriega et al^6^	2019	73 (80 vertebrae)	68 (75 vertebrae)	73.2	30:111	12 months
Tutton et al^10^	2015	141 (178 vertebrae)	144 (177 vertebrae)	75.5	74:211	12 months
Vanni et al^12^	2012	150	150	75	0:100	12 months
Werner et al^11^	2013	50	50	70	NA	“Post-operative”

3 studies reported outcomes for patients treated with the SpineJack system, while 2 reported on the Kiva® system and 1 compared traditional techniques to vertebral body stenting. All 6 studies reviewed patients who underwent kyphoplasty. Technique and device considered for comparison are outlined in Table 2, along with study conclusions.

**Table 2.** Comparison of techniques and conclusions among studies.

Author	Year	Device	Technique	Spinal level	Conclusion
Korovessis et al^4^	2013	Kiva®	Kyphoplasty	T6-L5 (Mostly T12, L1, L2)	Kiva® better-restored body wedge deformities and had lower cement leakage.
Noriega et al^2^	2016	SpineJack®	Kyphoplasty	T7-L3 (Mostly T11, T12,L1)	SpineJack® had a higher potential for vertebral body height restoration.
Noriega et al^6^	2019	SpineJack®, KyphX® Xpander®	Kyphoplasty	T7-L4 (Mostly T12, L1, L2)	MVPAs were non-inferior to kyphoplasty.
Tutton et al^10^	2015	Kiva®	Kyphoplasty	T1-L5 (Mostly T11, T12, L1)	Kiva® was non-inferior to kyphoplasty.
Vanni et al^12^	2012	SpineJack®	Kyphoplasty	T10-L5	SpineJack® was non-inferior to kyphoplasty.
Werner et al^11^	2013	Vertebral Body Stenting	Kyphoplasty	NA	Vertebral body stenting was associated with more material-related complications.

Risk of bias was evaluated using the RoB-2 Cochrane tool,^9^ as outlined in Table 3. 3 studies conveyed some concern regarding their methodology, while 3 studies were of low concern. The 3 studies of concern did not feature allocation concealment to study participants or blinding of outcome assessors.

**Table 3.** Risk of Bias 2 Cochrane tool for evaluating bias in randomised controlled studies. Risk of bias = high (+), low (-).

Study	Random Sequence Generation	Allocation Concealment	Blinding of Participants	Blinding of Outcome Assessors	Incomplete Outcome Data	Selective Reporting
Korovessis et al^4^	-	-	-	-	-	+
Noriega et al^2^	-	-	-	+	-	+
Noriega et al^6^	-	+	-	-	-	+
Tutton et al^10^	-	-	-	+	-	+
Vanni et al^12^	-	+	-	+	-	+
Werner et al^11^	-	+	-	+	-	+

### Operative Characteristics

(i) Operative Time (minutes)
  3 studies reported on operative time for both traditional techniques and MPVA systems.^2,6,12^ Noriega et al (2016) and Vanni et al (2012) found a significantly reduced operative time for procedures using MPVA systems (*P* = .001, *P* = <.05).^2,12^ Noriega et al^6^ (2019) found no significant difference (*P* = .175).
(ii) PMMA injected (mL)
  3 studies compared the quantity of PMMA (bone cement) used in traditional procedures vs those with MPVA systems.^4,6,10^ Each study reported significantly less PMMA injected when MPVA systems are used. However, this did not prove significant on collective analysis (RR = −1.46, CI = [-3.11;0.19]).

### Radiological Outcomes

**Figure 3.**
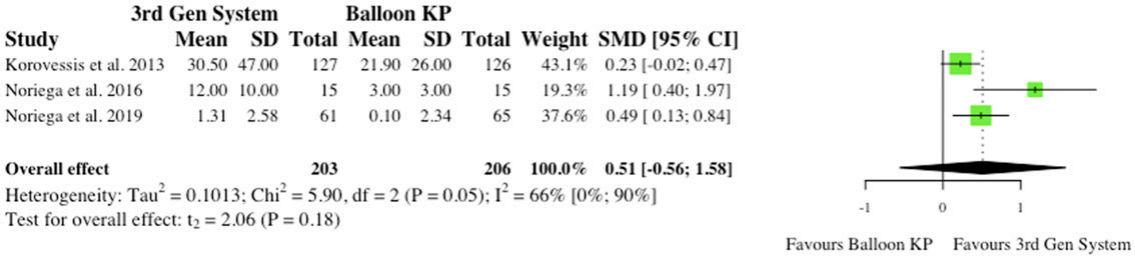
Midline vertebral body height ratio. *(SMD) = standardised mean difference. (95% CI) = 95% confidence interval. (SD) = standard deviation. (3rd Gen System = MPVA. (KP) = kyphoplasty.*

(i) Midline Vertebral Body Height Ratio
  3 studies reported the pre- to post-operative change in Midline Vertebral Body Height Ratio (MVBHr) among their patients.^2,4,6^ 2 studies found significant changes at 6 months post-operatively,^2,6^ but the overall effect was not statistically significant (RR = .51, CI = [-.56;1.58], *P* = .18) as seen in Figure 3.
(ii) Kyphosis and Gardner Angle
  2 studies reported changes in Kyphotic (Cobb) angle and Gardner angle pre to post-operatively. Thus, robust statistical analysis was deemed not to be appropriate. Noriega et al (2016) found significant reductions in Kyphotic angle following the use of MPVA systems (*P* = .012),^2^ while Werner et al (2013) did not (*P* = .972).^12^ Noriega et al (2016) and Korovessis et al (2013) found significant changes in both study groups, but inter-group significance at final follow-up was not determined.^2,4^

### Complications

**Figure 4.**
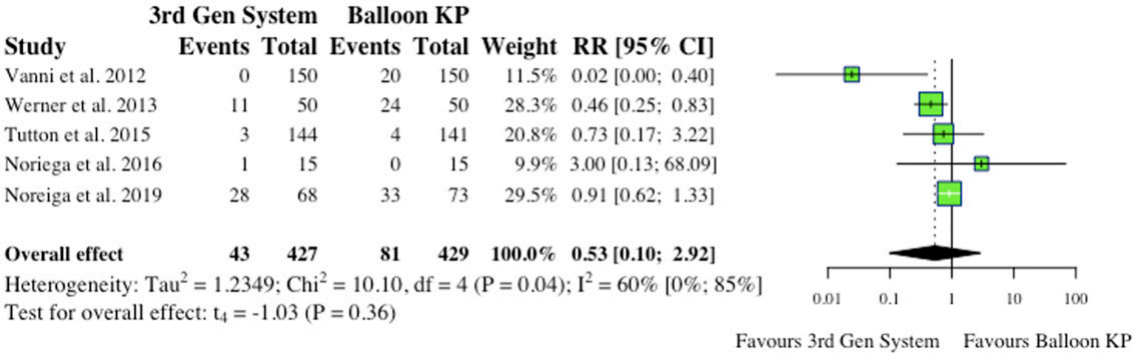
Total complications (inclusive of cement extravasation and device complications). *(RR) = relative risk. (95% CI) = 95% confidence interval. (3rd Gen System = MPVA). (KP) = kyphoplasty.*

**Figure 5.**
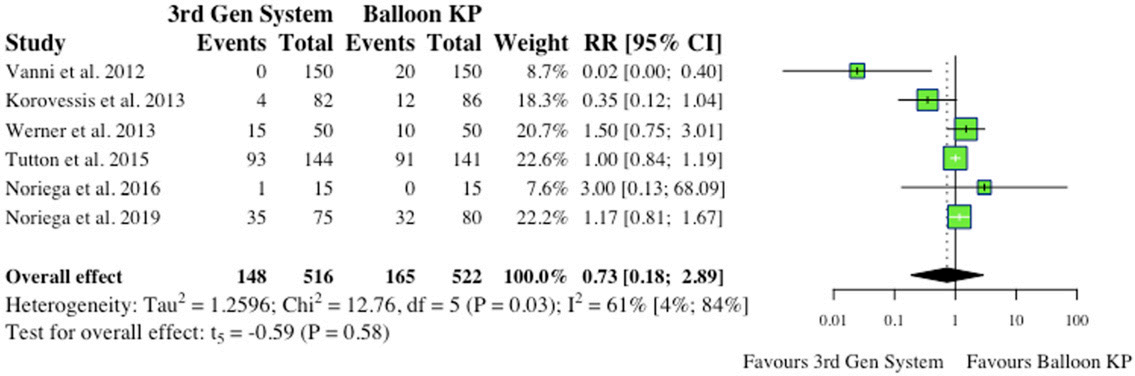
Cement extravasation.* (RR) = relative risk. (95% CI) = 95% confidence interval. (3rd Gen System) = MPVA. (KP) = kyphoplasty.*

**Figure 6.**
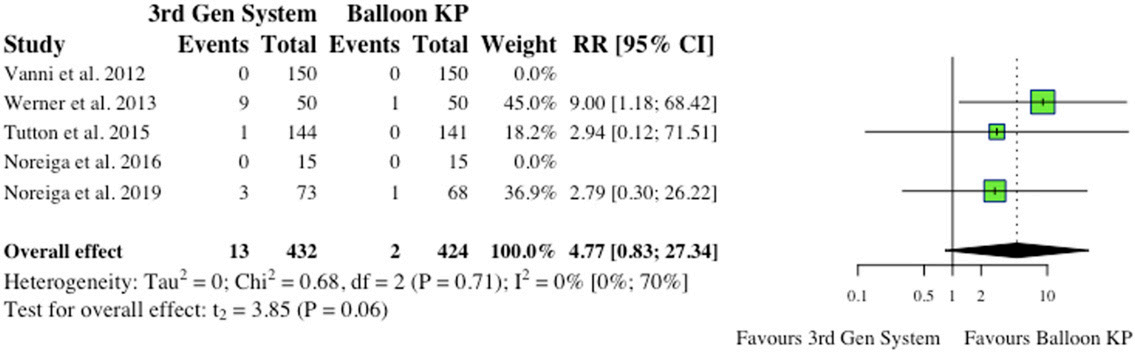
Device-related complications. *(RR) = relative risk. (95% CI) = 95% confidence interval. (3rd Gen System) = MPVA. (KP) = kyphoplasty. *

**Figure 7.**
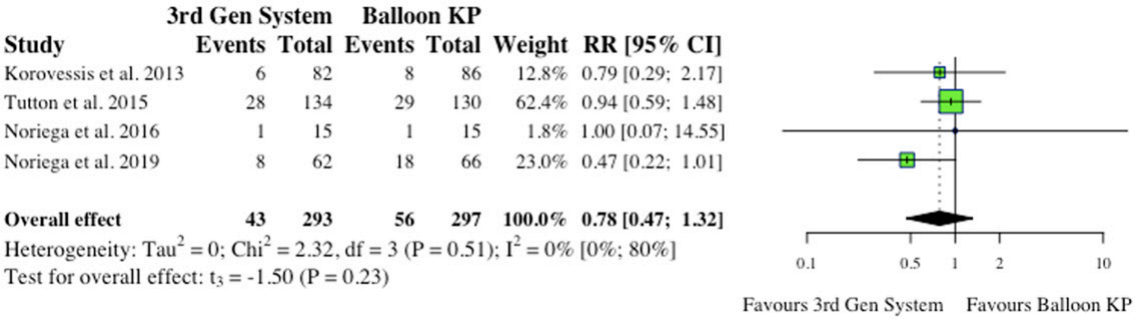
Adjacent fractures.* (RR) = relative risk. (95% CI) = 95% confidence interval. (3rd Gen System) = MPVA. (KP) = kyphoplasty. *

**Figure 8.**
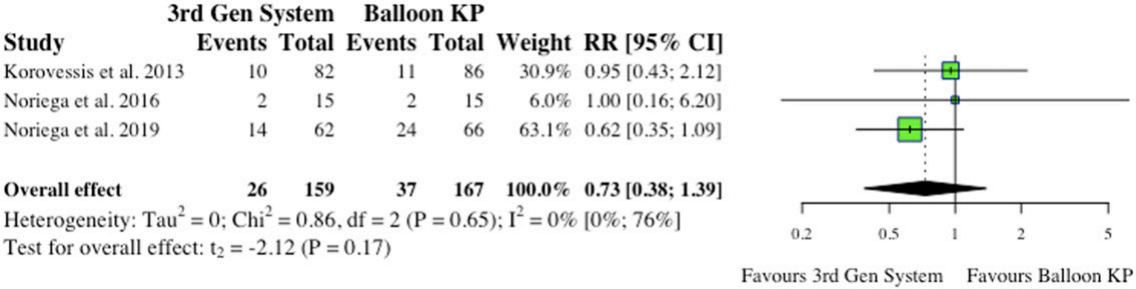
All new fractures.* (RR) = relative risk. (95% CI) = 95% confidence interval. (3rd Gen System) = MPVA). (KP) = kyphoplasty.*

(i) Total Complications
  5 studies reported total complications, which included both cement extravasation and device complications.^2,8,9,11,12^ Werner et al (2013) and Vanni et al (2012) found that significantly less complications occurred in their MPVA groups.^11,12^ The overall difference between groups was not statistically significant (RR = .53, CI = [.10;2.92], *P* = .36) as shown in Figure 4.
(ii) Cement Extravasation
  All 6 studies reported cement extravasation.^2,4,6,9,11,12^ While 1 found significant reductions in the MPVA group,^11^ the overall effect was not statistically significant (RR = .53, CI = [.13;2.17], *P* = .58), as depicted in Figure 5.
(iii) Device-Related Complications
  5 studies reported device-related complications.^2,6,9,11,12^ Typical complications reported include: death/life-threatening illness, events requiring hospitalization, permanent impairment of a bodily structure/function, requirement of medical/surgical intervention. As shown in Figure 6, the overall effect between study groups was not statistically significant (RR = 4.77, CI = [.83;27.34], *P* = .06).
(iv) Adjacent Fractures
  4 studies reported the occurrence of adjacent fractures.^2,4,6,10^ 3 of the 4 studies (75%) reported reduced incidence in the MPVA cohorts, as indicated in Figure 7. However, the overall effect was not statistically significant on collective analysis (RR = .78, CI = [.47;1.32], *P* = .23).
(v) All New fractures
  3 studies reported the incidence of all new fractures.^2,4,6^ 2 of 3 studies (66%) reported reduced incidence of all new fractures in the MPVA cohort. However, similarly to adjacent fracture analysis, the overall effect was not statistically significant on collective analysis (RR = .73, CI = [.38;1.39], *P* = .17), as shown in Figure 8.

### Functional Outcomes

**Figure 9.**
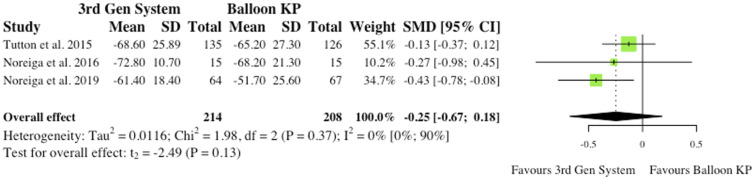
VAS change from baseline at 6 months. *(SMD) = standardised mean difference. (95% CI) = 95% confidence interval. (SD) = standard deviation. (3rd Gen System) = MPVA. (KP) = kyphoplasty.*

**Figure 10.**
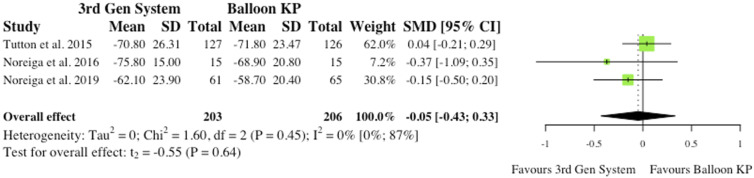
VAS change from baseline at final follow-up. *(SMD) = standardised mean difference. (95% CI) = 95% confidence interval. (SD) = standard deviation.*

**Figure 11.**
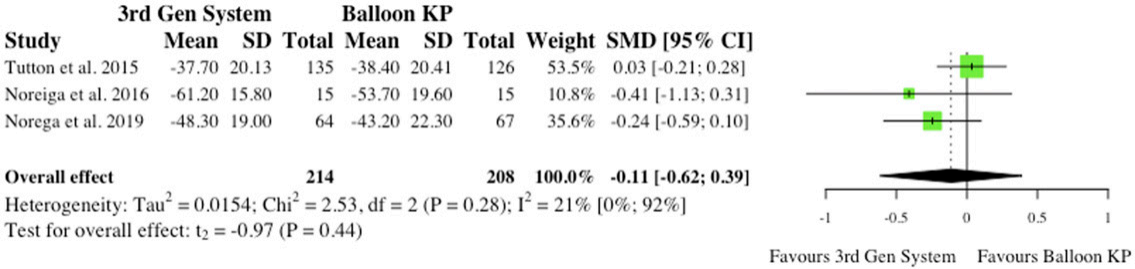
ODI change from baseline at 6 months.* (SMD) = standardised mean difference. (95% CI) = 95% confidence interval. (SD) = standard deviation. (3rd Gen System) = MPVA. (KP) = kyphoplasty.*

**Figure 12.**
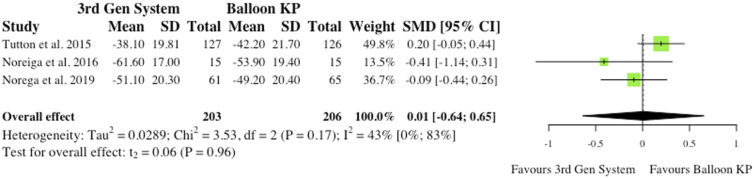
ODI change from baseline at final follow-up.* (SMD) = standardised mean difference. (95% CI) = 95% confidence interval. (SD) = standard deviation. (3rd Gen System) = MPVA. (KP) = kyphoplasty. *

(i) Visual Analogue Scale at 6-month Post-operatively
  3 studies reported Visual Analogue Scale (VAS) changes at 6 months post-operatively.^2,6,10^ Noriega et al (2019) found significantly lower scores in the MPVA group (*P* = .021).^6^ As reported in Figure 9, the overall effect was not statistically significant (SMD = −.25, CI = [-.67;0.18], *P* = .13).
(ii) Visual Analogue Scale at Final Follow-Up
  3 studies reported VAS changes at final follow-up.^2,6,10^ Results were contradictory as 2 studies report reduced VAS scores for MPVA systems at final follow-up, while 1 study favours traditional balloon KP. Understandably, the overall effect was therefore not statistically significant (SMD = −.05, CI = [-.43;0.33], *P* = .64), as seen in Figure 10.
(iii) Oswestry Disability Index at 6-month Post-operatively
  3 studies reported Oswestry Disability Index (ODI) changes at 6 months post-operatively.^2,6,10^ Similarly to VAS score, reduced disability burden in the MPVA group was evident in 2 studies, while 1 study saw reduced ODI scores in balloon KP cohort. As reported in Figure 11, the overall effect was not statistically significant (SMD = −.11, CI = [-.62;0.39], *P* = .44).
(iv) Oswestry Disability Index at Final Follow-Up
  3 studies reported ODI changes at final follow-up.^2,6,10^ Similarly to ODI scores at 6-month, the same 2 studies saw reduced ODI scores patients who received MPVA third generation systems, while 1 study favoured traditional balloon KP. The overall effect was not statistically significant (SMD = .01, CI = [-.64;0.65], *P* = .96) (Figure 12).

## Discussion

As the population steadily ages worldwide, osteoporosis continues to grow as a major health concern.^1^ It is well described in the literature that factors such as age, gender, lifestyle, medication usage and autoimmune disease play a role in the balance between bone formation and resorption. When these factors are skewed, individuals become susceptible to the development of osteoporosis and are more likely to experience OVCFs, leading to increased morbidity and possibly mortality, with a predilection for the elderly population.^13^ As mentioned, conservative treatment measures for these individuals include bed rest, physical therapy, exercise, bracing, oral medications and injections.^14^ Ultimately, operative methods are considered when these conservative measures fail to provide adequate pain relief and restoration of function. Traditionally, operative techniques such as kyphoplasty and vertebroplasty have been utilised to improve patient outcomes. However, with these procedures come major disadvantages, such as incomplete fracture reduction and post-operative loss of height restoration through cement leakage.^4^ Consequently, MPVA systems were introduced as an alternative to traditional techniques in hopes of improving functional outcomes and minimising post-operative complications. Despite their increased use, there is little research stating a clear benefit of MPVAs with statistically significant improvement when comparing clinical and radiological study variables. Although reported complications rates are low,^12^ systematic research and meta analyses are needed to warrant such an increase in usage. As prevalence of osteoporosis rises along with the aging population,^1^ ensuring the safety and efficacy of MPVA devices is an important and necessary topic within spine research.

Studies included in this review directly compared MPVAs to traditional methods of BKP, reporting patient data for a variety of measures such as operative characteristics, radiological outcomes, complications and functional outcomes. Due to this, these studies are useful to determine whether MPVAs provide a true statistically significant benefit over kyphoplasty and vertebroplasty. Regarding operative characteristics, Noriega et al (2016) and Vanni et al (2012) found a significantly reduced operative time for procedures using MPVA systems (*P* = .001, *P*=<.05).^2,12^ It is well known that in order to achieve favourable patient outcomes, institutions that perform spinal surgeries must have rigorous protocols in place and a high level of institutional experience to minimise infection risk and improve patient outcomes.^15^ These enhanced protocols can often come with a prolongation of operating times, which, in itself, are associated with an increase in potential risks to patients.^16^ The reduction in operating time for procedures using MPVA systems that this review has identified is a potential advantage over conventional methods to improve patient outcomes.

Furthermore, several studies reported the amount of PMMA (bone cement) used in traditional procedures vs those with MPVA systems^4,6,10^; each of these studies reported significantly less PMMA injected when MPVA systems were used. Within the literature, it is well described that the major complications in traditional procedures to treat OVCFs include cement extravasation, fractures, and device-related complications with cement leakage being a particular concern following vertebroplasty procedures.^16^ It is also noted that predictive factors for adjacent fractures include the kyphotic angle, cement distribution and grade of disc degeneration. While most cases of cement leakage are clinically asymptomatic, leakage into the intervertebral disc space has been reported as a risk factor for increased incidence of fracture in the adjacent vertebrae; with the direct relationship between the amount of cement leakage to an increased fracture risk remaining unclear.^17-19^ Additionally, cement leakage into the spinal canal has also been linked as a causative factor for post-operative canal stenosis and spinal cord compression.^17^ Each of the 6 studies included reported cement extravasation in kyphoplasty and vertebroplasty procedures or those using MPVAs.^2,4,6,10-12^ While 1 reported significant reductions in the MPVA group,^12^ the overall effect was not statistically significant. Similar results were found for device-related complications. Werner et al (2013) and Vanni et al (2012) found that significantly less total complications (device plus cement extravasation) occurred in their MPVA groups.^11,12^ However, the net effect between groups failed to determine the difference was significant. Studies reporting fracture incidence post-operatively also failed to prove MPVA superiority. Therefore, when considering complication rates there is a lack of clear benefit for MPVA systems over traditional operative methods.

When considering radiological outcomes, we reviewed the findings of 4 studies included in this review.^2,4,6,10^ 3 of these studies explored post-operative MVBHr changes, with no statistically significant effect overall.^2,4,6^ Noriega et al^2^ (2016) found that MPVA systems provided significant reductions in Cobb angle . Korovessis et al^4^ (2013) also found significant radiological changes in both study groups . However, inter-group significance was not determined at final follow-up. Additionally, insufficient data was available to reasonably compare the change in Gardner angle between groups; which proved to be a limitation of this study.

Functional outcomes were mainly assessed using the VAS and ODI scoring systems. 3 studies included in this review reported significant changes in both the mean VAS and ODI in their MPVA patient populations at final follow-up.^2,6,10^ The overall effect, however, failed to show a significant improvement in comparison to kyphoplasty patients. A study by Ender et al^20^ (2013) determined clinical and radiological outcomes plus complication rates in OVCF patients treated using the OsseoFix® system. Their findings showed significant improvements across all parameters, including the mean VAS, mean ODI, kyphotic angle and occurrence of complications such as adjacent fractures. This study highlights the existing literature which supports the safety and efficacy of MPVAs, but lacks a comparative aspect to endorse a functional benefit over traditional procedures.

Overall, the findings of this study support the use of MPVA systems, as notable clinical and radiological improvements were observed in OVCF patients. However, this statistical analysis failed to show a clear benefit when comparing MPVAs to the traditional surgical techniques such as balloon kyphoplasty. This, along with capital cost of instrumentation ultimately questions the superiority of MPVAs over traditional cement augmentation techniques.^21-25^ It is worth noting that when newer techniques, procedures, and technologies are released, nonclinical reasons for their implementation are often rooted in their marketing, administrative pressure and peer pressure within the field^26^; which has the potential to lead to their hastened and unnecessary adoption without statistical evidence proving their superiority. These points lament the need for additional evidence with long-term follow-up data to fully understand the potential advantages or disadvantages to adopting innovative techniques such as MPVAs. Alternatively, there may be potential for a regenerative biomaterial in the treatment of OVCF rather than cement or extravasation. However, no robust evidence exists to date.

Notably, this review does have limitations. The most notable being heterogeneity between studies included and the limited quantity of literature which fit the inclusion criteria. The heterogeneity between the studies included exists for many reasons. The differences of time-points in which study parameters were evaluated post-operatively by the various authors included proved difficult to analyse through a meta-analysis. Additionally, the studies included were not uniform in their reporting of patient characteristics and outcomes, and, therefore, this led to an inability to analyse key subgroups through this meta-analysis. In order to fully convey the evidence necessary for practicing spine surgeons to make an informed decision in OVCF management, subgroup analysis comparing these procedures within older vs younger patient populations, comparing the time frame of intervention (immediately after trauma vs at a distance), and comparing the potential influence of medical treatment pre-operatively on post-operative outcomes must be thoroughly explored and reported. Moreover, literature for consideration was restricted due to the need for comparative studies. While the findings included in this review provided an adequate volume of data to complete a statistical analysis, we found that many areas of our meta-analysis that trended towards MPVAs being the superior option for treatment lacked statistical significance. Therefore, 1 limitation for this review is the limited number of studies included as this has the potential to skew the statistical power of our results. In regard to future research, additional studies focused on evaluating the usage of MPVAs compared to traditional techniques are necessary to elucidate whether these novel devices improve post-operative outcomes in osteoporotic patients with vertebral compression fractures.

## Conclusion

All in all, it is well known that osteoporotic compression fractures remain an injury with a high associated morbidity and profile within spinal surgery. The purpose of this review was to compare postoperative outcomes for new third generation augmentation systems vs traditional cement augmentation techniques to determine whether the novel MPVA devices could improve OVCF outcomes. The results of this meta-analysis highlight no significant improvement in clinical or radiological outcomes for MPVA systems when compared to balloon kyphoplasty for vertebral body augmentation. This study is limited by the heterogeneous, limited size of our patient population and the consequent inability to conduct subgroup analysis across the population. Further research is needed to establish a true benefit of MPVA systems over traditional operative methods.
